# *DIRC3* and near *NABP1* genetic polymorphisms are associated laryngeal squamous cell carcinoma patient survival

**DOI:** 10.18632/oncotarget.12865

**Published:** 2016-10-25

**Authors:** Zhen Shen, Wanli Ren, Yanxia Bai, Zhengshuai Chen, Jingjie Li, Bin Li, Tianbo Jin, Peilong Cao, Yuan Shao

**Affiliations:** ^1^ Department of Otolaryngology & head neck, The First Affiliated Hospital of Xi'an Jiaotong University, Xi'an, Shaanxi 710061, China; ^2^ Department of Pathology, The First Affiliated Hospital of Xi'an Jiaotong University, Xi'an, Shaanxi 710061, China; ^3^ School of Life Sciences, Northwest University, Xi'an, Shaanxi 710069, China; ^4^ National Engineering Research Center for Miniaturized Detection Systems, Xi'an 710069, China

**Keywords:** laryngeal squamous cell carcinoma, NABP1, DIRC3, biomarker, polymorphism

## Abstract

Laryngeal squamous cell carcinoma (LSCC) is one of the most common and aggressive malignancies of the upper digestive tract. The present study is a retrospective analysis of data from a prospective longitudinal study. A total of 170 male LSCC patients (average age, 60.75±10.082) at the First Affiliated Hospital of Xi'an Jiaotong University School of Medicine were recruited between January 2002 and April 2013 for this study. We assessed correlations between patient characteristics and survival, and sequenced genomic DNA from patient peripheral blood samples. We found that the single nucleotide polymorphisms (SNPs), rs11903757, with closest proximity to *NABP1* and *SDPR*, and rs966423 in *DIRC3*, were associated with survival in LSCC patients. Median follow-up was 38 months (range 3–122) and median survival time was 48 months. LSCC patients with total laryngectomy, poor differentiation, T3-T4 stage, N1-N2 stage or III-IV TNM stage had reduced survival. This is the first study to demonstrate that the rs11903757 GT (HR=2.036; 95% CI, 1.071–3.872; *p*=0.030) and rs966423 TT (HR=11.677; 95% CI, 3.901–34.950; *p*=0.000) genotypes predict poor patient outcome. These polymorphisms may serve as useful clinical markers to predict patient survival, and to guide individual patient therapeutic decisions.

## INTRODUCTION

Carcinomas of the upper aerodigestive tract represent a major challenge in modern health care. Laryngeal squamous cell carcinoma (LSCC), which directly impacts patient speech and communication, is one of the most common and aggressive malignancies of the upper digestive tract [1, 2]. For LSCC patients, poor early diagnosis rates result in delayed treatment and higher levels of disease recurrence and metastasis.

In China, LSCC incidence has been rising gradually, especially in the northeast. Despite significant advances in surgery and radio-therapeutic techniques and new chemotherapeutics, the 5-year relative survival rate for LSCC patients has not markedly improved, and mortality is still high at 1.2 cases per 100,000 persons [6]. Additionally, there are currently no ideal prognostic biomarkers to guide laryngeal cancer patient treatment. Presently, much work is focused on the identification of useful diagnostic and therapeutic markers [7, 8], and recent findings suggest that the combined expression patterns of multiple genes may be useful prognostic indicators. Tissue microarray and proteomics technologies have enabled the discovery of LSCC metastasis suppressor genes, which have strong potential for use as biomarkers. Still, high morbidity and low cure rates necessitate the development of new and improved diagnostic procedures, biomarkers and therapeutics to effectively treat LSCC [3–5].

The present study is a retrospective analysis using data from a prospective longitudinal study of 170 patients over an extended time period (2002–2013). We examined LSCC patient epidemiology with regard to age, laryngectomy, neck dissection, tumor differentiation, T-stage, N-stage, TNM stage and treatment modality. In analyzing possible genetic polymorphisms associated with LSCC susceptibility and prognosisS, we hoped to identify possible diagnostic markers or therapeutic targets that could lead to improved patient survival.

## RESULTS

### Patient characteristics and treatment outcomes

**Figure 1:**
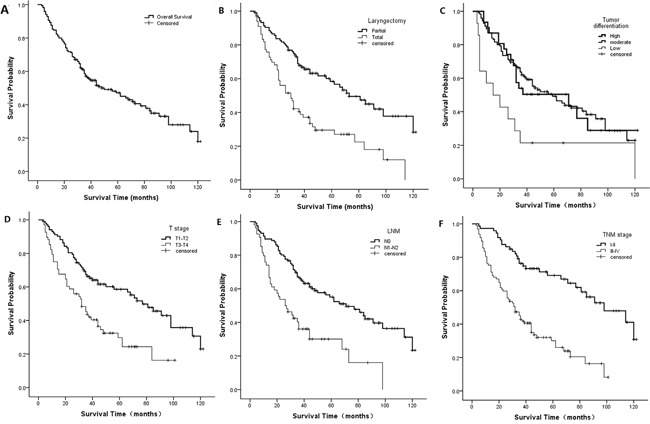
Kaplan-Meier curves for potential factors influencing LSCC patient survival Overall survival **A.** laryngectomy (*p*=0.000, log-rank test) **B.** Tumor differentiation (*p*=0.008, log-rank test) **C.** T-stage (*p*=0.000, log-rank test) **D.** N-stage (*p*=0.000, log-rank test) **E.** TNM stage (*p*=0.000, log-rank test) **F.** Curves represent 10 years of follow-up.

A total of 170 LSCC patients at the First Affiliated Hospital of Xi'an Jiaotong University School of Medicine were recruited from January 2002 and April 2013 for this study. All patients were males with an average age of 60.75±10.082 years (mean ± SD) (range, 32–82 years). Patients were followed up from diagnosis until the end of April, 2013. No patient was lost from follow-up data. The median follow-up time was 38 months (range, 3–122 months) and median survival time was 48 months. 100/170 (58.82%) patients died of LSCC during the study period. The 5-year overall survival rate was 47.1% (Figure 1A). Patient deaths were regarded as censored data and were so marked on survival curves. Characteristics of the 14 candidate single nucleotide polymorphisms (SNPs) assessed in this study are shown in Table 1.

**Table 1:** Basic information of SNPs in this study

SNP-ID	Position	Band	Allele	Gene	Role
rs17401966	10385471	1p36.22	G/A	KIF1B	Intron
rs4072037	155162067	1q22	G/A	MUC1	Coding exon
rs1912453	162821291	1q23.3	C/T	C1orf110	Downstream
rs10911251	183081194	1q25.3	A/C	LAMC1	Intron
rs6687758	222164948	1q41	G/A	−	−
rs11903757	192587204	2q32.3	C/T	−	−
rs9288520	217481271	2q35	A/G	−	−
rs966423	218310340	2q35	T/C	DIRC3	Intron
rs6736997	235615197	2q37.2	A/C	−	−
rs975334	2846316	3p26.2	C/T	CNTN4	Intron
rs8180040	47388947	3p21.31	A/T	KLHL18	Downstream
rs9841504	114362764	3q13.31	G/C	ZBTB20	Intron
rs10936599	169492101	3q26.2	T/C	ARPM1	Promoter
rs2239612	186793242	3q27.3	T/C	ST6GAL1	Intron

Potential factors that influenced the prognosis are shown in Table 2. We investigated relationships between these factors and survival by univariate analysis, and survival curves were drawn using the Kaplan-Meier method. We found that patient laryngectomy (*p*=0.000, Figure 1B), differentiation (*p*=0.008, Figure 1C), T-stage (*p*=0.000, Figure 1D), N-stage (*p*=0.000, Figure 1E), and TNM stage (*p*=0.000, Figure 1F) were associated with survival based on the log-rank test. However, survival was not correlated with age (*p*=0.456) and neck dissection (*p*=0.188) in stratified analyses. Compared with partial laryngectomy, high differentiation, T1–T2 stage, N0 stage and I–II TNM stage, the HR for total laryngectomy (95% CI, 1.576–3.492; *p*=0.000), poor differentiation (95% CI, 1.150–4.997; *p*=0.020), T3-T4 stage (95% CI, 1.448–3.253; *p*=0.000), N1-N2 stage (95% CI, 1.582–3.623; *p*=0.000) and III-IV TNM stage (95% CI, 2.100–5.180; *p*=0.000) increased to 2.346, 2.397, 2.170, 2.394 and 3.298, respectively.

**Table 2:** Patient characteristics and treatment outcomes - univariate associations with LSCC prognosis

	N (Total/Events)	Median	*P*-value^a^	HR (95% CI)	*P*-value^b^
**Age (years)**					
<60	80/47	59	0.456	1	
≥60	90/53	48		1.161 (0.782 - 1.722)	0.460
**Laryngectomy**					
Partial	104/50	73	0.000*	1	
Total	66/50	30		2.346 (1.576 - 3.492)	0.000*
**Neck dissection**					
yes	37/19	36	0.188	1	
no	133/81	56		0.711 (0.42 - 1.189)	0.194
**Differentiation**					
high	31/18	71	0.008*	1	
moderate	125/70	59		0.933 (0.556 - 1.567)	0.794
poor	14/12	15		2.397 (1.150 - 4.997)	0.020*
**T-stage**					
T1-T2	102/52	77	0.000*	1	
T3-T4	68/48	32		2.170 (1.448 - 3.253)	0.000*
**N-stage**					
N0	116/61	71	0.000*	1	
N1-N2	54/39	26		2.394 (1.582 - 3.623)	0.000*
**TNM stage**					
I-II	73/30	98	0.000*	1	
III,IV	97/70	32		3.298 (2.100 - 5.180)	0.000*

a **Notes:** *P*-values based on the log-rank test;

b *P*-values based on the Wald test;

* p-value < 0.05 indicates statistical significance.

**Abbreviations:** HR, hazard ratios; CI: confidence intervals.

### Genetic polymorphisms and outcome correlations

**Figure 2:**
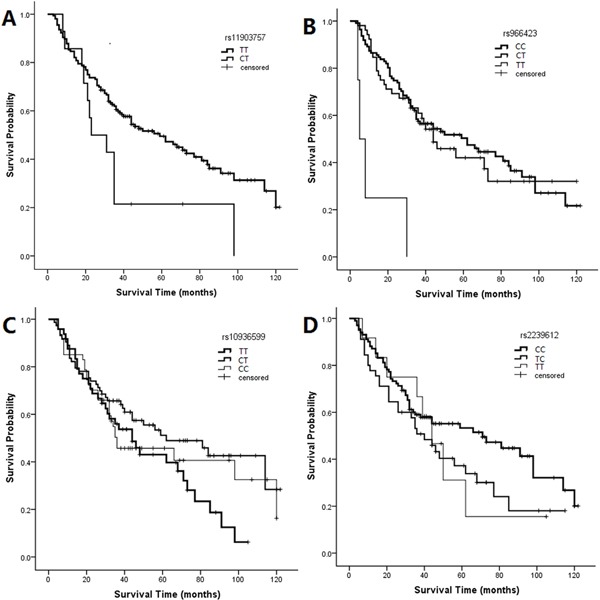
Kaplan-Meier curves for SNPs associated with LSCC patient survival rs11903757 genotypes (*p*=0.021, log-rank test) **A.** rs966423 genotypes (*p*=0.000, log-rank test) **B.** rs10936599 genotypes (*p*=0.153, log-rank test) **C.** rs2239612 genotypes (*p*=0.192, log-rank test) **D.** Graphs represent 10 years of follow-up.

We assessed the associations between 4 genotypes and survival by univariate analysis (Table 3). Two SNPs (rs11903757, *p*=0.021, Figure 2A; rs966423, *p*=0.000, Figure 2B) were associated with survival when all patients were examined using the log-rank test. For rs11903757 near *NABP1*, the CT genotype (HR, 2.001; 95% CI, 1.091–3.673; *p*=0.025) resulted in a higher risk than the TT genotype. With regard to rs966423 in *DIRC3*, survival was worse in patients with the TT genotype (HR, 7.721; 95% CI, 2.748–21.695; *p*=0.000) compared to those with the CC genotype. The TC genotype (HR, 1.089; 95% CI, 0.701–1.690; *p*=0.705) was not associated with survival.

**Table 3:** Genetic polymorphisms and outcome - univariate associations with LSCC prognosis

SNP-ID	genotype	N(Total/Events)	Median	*P*-value^a^	HR (95% CI)	*P*-value^b^
rs17401966	A/A	125/73	48.0		1	
	G/A	36/19	66.0	0.437	0.905 (0.545 - 1.502)	0.669
	G/G	8/7	35.0		1.576 (0.724 - 3.430)	0.252
rs4072037	A/A	16394	46		1	
	G/A	2/2	81	0.843	0.976 (0.239 - 3.985)	0.973
	G/G	3/3	59		1.403 (0.442 - 4.450)	0.565
rs1912453	T/T	41/25	46		1	
	C/T	113/65	62	0.389	0.936 (0.589 - 1.486)	0.779
	C/C	15/10	35		1.489 (0.712 - 3.116)	0.290
rs10911251	C/C	45/28	36		1	
	A/C	81/48	59	0.720	0.831 (0.518 - 1.333)	0.443
	A/A	34/19	66		0.949 (0.530 - 1.700)	0.860
rs6687758	A/A	114/68	59		1	
	G/A	44/26	44	0.979	1.047 (0.664 - 1.651)	0.842
	G/G	5/2	34		0.975 (0.238 - 4.000)	0.972
rs11903757	T/T	156/88	59		1	
	C/T	14/12	23	0.021*	2.001 (1.091 - 3.673)	0.025*
	C/C	−/−	−		−	−
rs9288520	G/G	92/52	48		1	
	A/G	72/45	50	0.786	1.085 (0.727 - 1.619)	0.691
	A/A	4/2	22		0.692 (0.166 - 2.877)	0.613
rs966423	C/C	111/65	62		1	
	T/C	52/29	44	0.000*	1.089 (0.701 - 1.690)	0.705
	T/T	4/4	5		7.721(2.748 - 21.695)	0.000*
rs6736997	C/C	105/66	44		1	
	A/C	64/33	62	0.540	0.878 (0.577 - 1.336)	0.543
	A/A	−/−	−		−	−
rs975334	T/T	125/74	48		1	
	C/T	42/25	46	0.584	1.017 (1.017 - 1.603)	0.941
	C/C	3/1	59		0.373 (0.052 - 2.687)	0.328
rs8180040	T/T	51/32	44		1	
	A/T	51/28	62	0.754	0.836 (0.501 - 1.394)	0.493
	A/A	43/24	62		0.858 (0.505 - 1.458)	0.572
rs9841504	G/G	132/77	56		1	
	G/C	26/16	40	0.680	1.120 (0.652 - 1.925)	0.682
	C/C	3/2	26		1.763 (0.430 - 7.223)	0.431
rs10936599	C/C	48/34	44		1	
	T/C	73/37	62	0.153	0.635 (0.398 - 1.014)	0.057
	C/C	47/28	36		0.818 (0.494 - 1.356)	0.436
rs2239612	C/C	101/54	71		1	
	T/C	45/31	40	0.192	1.488 (0.954 - 2.321)	0.080
	T/T	12/8	44		1.332 (0.632 - 2.810)	0.451

a **Notes:** *P*-values based on the log-rank test;

a *P*-values based on the Wald test;

* p-value < 0.05 indicates statistical significance.

**Abbreviations:** HR, hazard ratios; CI: confidence intervals.

### Multivariate analysis of treatment outcome

We hypothesized that the rs11903757 and rs966423 genotypes might be prognostic markers in LSCC. Univariate analysis showed that patients with total laryngectomy, poor differentiation, T3–T4 stage, N1–N2 stage, III–IV TNM stage or these SNPs had worse survival. This suggests that rs10936599 (*p*=0.153, Figure 2C) in the promoter region of *ARPM1* and rs2239612 (*p*=0.192, Figure 2D) in the intron region of *ST6GAL1* might be associated with prognostic survival. We adjusted for the above confounding factors in a multivariate Cox proportional hazards model, calculating HR and 95% CI for different genotypes, to measure the impacts of alleles on prognosis (Table 4). With regard to rs11903757, the genotype CT (HR, 2.036; 95% CI, 1.071–3.872; *p*=0.030) increased the HR more than two-fold compared to the TT genotype. For rs966423, survival was worse in patients with the TT genotype (HR, 11.677; 95% CI, 3.901–34.950; *p*=0.000) than those with the CC genotype. Rs10936599 and rs2239612 were not related to prognosis.

**Table 4:** Genetic polymorphisms and outcome - multivariate associations with LSCC prognosis

SNP-ID	Genotype	HR(95% CI)	*P*-value
rs11903757	TT	1	
	CT	2.036(1.071 - 3.872)	0.030*
rs966423	CC	1	
	TC	0.154(0.727 - 1.833)	0.543
	TT	11.677(3.901 - 34.950)	0.000*
rs10936599	TT	1	
	CT	0.780(0.475 - 1.280)	0.326
	CC	0.933(0.555 - 1.567)	0.792
rs2239612	CC	1	
	TC	1.232(0.779 - 1.949)	0.373
	TT	1.224(0.576 - 2.600)	0.599

**Notes:** ^b^*P*-values based on the Wald test;

* p-value < 0.05 indicates statistical significance.

**Abbreviations:** HR, hazard ratios; CI: confidence intervals.

## DISCUSSION

Laryngeal cancer is the second most common type of head and neck cancer. An estimated 12,000 new laryngeal cancer cases are diagnosed in the USA every year. The incidence of laryngeal cancer is much higher in men than in women, especially for those between 60 and 70 years of age [6]. All patients in the current study were men. Consistent with other studies, we found that patients with total laryngectomy, poor differentiation, T3–T4 stage, N1–N2 stage or III–IV TNM stage had reduced survival [9–12]. We developed multivariate Cox regression analysis models that adjusted for the most important covariates, including SNP, tumor differentiation, neck dissection, T stage and TNM stage. In some researches indicated that patients T-stage and N-stage had demonstrated to be significantly associated with survival and these findings are consistent with results of most other studies. Our study also obtained the consistent conclusion [9, 13, 14]. A multivariate analysis was conducted, taking the variables found to be significant in univariate analyses (*p*≤0.2) into account. We showed that rs11903757 and rs966423 polymorphisms correlate with LSCC patient survival. In particular, theS rs11903757 CT and rs966423 TT genotypes correlated with poorer outcomes in this patient group.

Rs11903757 is an intergenic SNP on chromosome 2q32.3 with closest proximity to *NABP1* (44 kb centromeric) and *SDPR* (112 kb telomeric), which encodes the serum-deprivation response phosphatidylserine-binding protein [15]. The CT genotype was associated with reduced survival. Peters, et al. [16] recently reported a genome-wide association between rs11903757 and colorectal cancer risk in a combined analysis of European and Asian case-control studies. However, another study [17] failed to corroborate previously published data showing an association between rs11903757 and colorectal cancer risk.

Rs966423 is located in the *DIRC3* gene intron region at 2q35 and the TT genotypes were associated with worse survival in SCLC patients. *DIRC3* (disrupted in renal cancer 3) was first identified in 2003. Its disruption by a t(2;3) (q35;q21) translocation was observed in renal cell carcinoma [18], and although the function of *DIRC3* is unknown, it is presumed to have tumor suppressor activity. In a genome-wide association study, *DIRC3* was associated both with thyroid cancer risk and thyroid stimulating hormone level [19]. It is thus possible that *DIRC3* changes alter thyroid stimulating hormone production and, indirectly, promote thyroid cancer development as a result of decreased thyroid epithelium differentiation. Additional research is needed to determine whether or not a similar pathway promotes LSCC. The rs966423 TT genotype was reportedly associated with increased overall mortality in patients with differentiated thyroid cancer [20]. The rs966423 [20]. The CT and CT + TT genotypes were more common in papillary thyroid cancer patients with extra-thyroidal extension and more advanced T stage [21–23]. Laryngeal and thyroid carcinoma are the two main malignant tumor types of the head and neck, and both sides of the thyroid gland attach to the lower part of the throat. Thyroid carcinomas were incidentally found in 0.7–3% of surgeries for another primary head and neck cancer of non-thyroid origin [24–29]. A clinically unexpected, simultaneous thyroid cancer confirmed postoperatively from thyroid tissue partially removed with a laryngeal cancer specimen is rare [30], and these findings require additional confirmation.

Our research had some limitations. First, our sample size of 170 patients was small, and a wider sample range is needed to verify our results. Second, our samples were geographically limited to Shaanxi, China, and a larger number of samples from different ethnic populations must be studied. In addition, our study did not elucidate the functional relevance of the variants to gain insight into the mechanisms underlying the association.

In conclusion, this study showed that LSCC patients with total laryngectomy, poor differentiation, T3–T4 stage, N1–N2 stage or III–IV TNM stage had reduced survival and was the first to demonstrate that the rs11903757 GT and rs966423 TT genotypes predict poor patient outcome. These polymorphisms may serve as useful clinical markers to predict patient survival, and to guide individual patient therapeutic decisions.

## MATERIALS AND METHODS

### Ethics approval and patient consent

Informed consent was obtained from each patient according to protocols approved by the ethics committees of the First Affiliated Hospital of Xi'an Jiaotong University School of Medicine.

### Subjects

We identified 170 patients without distant LSCC metastasis who underwent partial or total laryngectomy. Patient blood samples were randomly collected at the First Affiliated Hospital of Xi'an Jiaotong University School of Medicine from January 2002 to April 2013. Histologic tumor diagnosis was made and agreed upon by at least two senior pathologists at the Department of Pathology based on World Health Organization (WHO) criteria. All patients were men aged 32 to 82 years with an average age of 60.75 years. X-rays, computed tomography (CT) scans, laryngoscopy, examination of laryngeal lesions, local cell smears and pathology examinations were used for diagnosis. Eligible patients had pathologically-confirmed laryngeal carcinoma without distant metastases (M0). All patients underwent a standard clinical examination within two months of diagnosis and none had received any therapy before admission for surgery. All cases were systematically classified based on the Union of International Cancer Control (UICC, 2010) TNM staging system of laryngeal carcinomas, which essentially establishes the modality of therapy.

Follow-up included access to medical records and telephone contact. Patient medical records were reviewed to assess patient characteristics, including age, laryngectomy (partial or total), neck dissection (yes or no), tumor differentiation, T-stage, N-stage, TNM stage and final status on the last follow-up examination. We chose April 2013 as the eligibility end time with the goal of having adequate follow-up for individual participants, with a median follow-up time of 48 months (range, 3–122 months). 100 patients died during the study period and no patient was lost from follow-up data. Survival time was defined as from the date of surgery to the date of death.

### SNP selection and genotyping

We selected 14 total SNPs in chromosomes 1, 2 and 3 that had minor allele frequencies (MAF) >5% and were associated with cancer of the aerodigestive tract in the HapMap Asian population. We extracted genomic DNA from peripheral blood using a GoldMag-Mini Whole Blood Genomic DNA Purification Kit (GoldMag Ltd. Xi'an, China) according the manufacturer's protocol. SNP genotypes were obtained according to the standard protocol recommended by Sequenom MassARRAY RS1000 [31]. Finally, Sequenom Typer 4.0 Software was used for data management and analysis [31, 32].

### Statistical analysis

Correlations between categoric variables were assessed using the chi-square test. Survival curves were drawn using the Kaplan-Meier method. Differences between curves were analyzed using the log-rank test. A Cox proportional hazards model was applied to estimate risk by calculating hazard ratios (HR) and 95% confidence intervals (CI) for categorical variables of exposure. Multivariate Cox regression analysis models were then developed that adjusted for the most important covariates. The SPSS statistical software package version 17.0 (SPSS Inc., Chicago, IL, USA) was used for all analyses. *P*<0.05 was considered statistically significant.
